# Supplementary material for: Lipid Nanoparticle Database towards structure-function modeling and data-driven design for nucleic acid delivery
Source: Nat Commun. 2026 Jan 28;17:2464. doi: 10.1038/s41467-026-68818-1 (PMC12992592; doi:10.1038/s41467-026-68818-1)
Supplement: Supplementary file 1 — Supplementary Information [file 41467_2026_68818_MOESM1_ESM.pdf]

Supplementary Information

Lipid Nanoparticle Database towards structure-function modeling and data-driven design for nucleic acid delivery

Evan Collins[1,2,3,*], Jungyong Ji[4,*], Sung-Gwang Kim[4,*], Jacob Witten[1,2,5], Seonghoon Kim[4], Richard Zhu[6], Peter Park[7], Minjun Jung[4], Aron Park[4], Rajith S. Manan[2,5], Arnab Rudra[2,5,8], Gyochang Keum[9], Eun-Kyoung Bang[9,10], Jun-O Jin[11], William J. Jeang[2,8,12], Robert Langer[1,2,5,13,14], Daniel G. Anderson[2,5,8,13,14,†], Wonpil Im[4,7,†]

[1]Department of Biological Engineering, Massachusetts Institute of Technology, Cambridge, MA, USA
[2]David H. Koch Institute for Integrative Cancer Research, Massachusetts Institute of Technology, Cambridge, MA, USA
[3]Jameel Clinic, Massachusetts Institute of Technology, Cambridge, MA, USA
[4]MolCube Inc., Seoul, Republic of Korea
[5]Department of Chemical Engineering, Massachusetts Institute of Technology, Cambridge, MA, USA
[6]Department of Biology, Massachusetts Institute of Technology, Cambridge, MA, USA
[7]Department of Biological Sciences, Lehigh University, Bethlehem, PA, USA
[8]Department of Anesthesiology, Critical Care and Pain Medicine, Boston Children's Hospital, Boston, MA, USA
[9]Medicinal Materials Research Center, Biomedical Research Division, Korea Institute of Science and Technology, Seoul, Republic of Korea
[10]KHU-KIST Department of Converging Science and Technology, Graduate School, Kyung Hee University, Seoul, Republic of Korea
[11]Department of Microbiology, Brain Korea 21 Project, University of Ulsan College of Medicine, ASAN Medical Center, Seoul, Republic of Korea
[12]Department of Materials Science and Engineering, Massachusetts Institute of Technology, Cambridge, MA, USA
[13]Harvard and MIT Division of Health Science and Technology, Massachusetts Institute of Technology, Cambridge, MA, USA
[14]Institute for Medical Engineering and Science, Massachusetts Institute of Technology, Cambridge, MA, USA
[*]These authors contributed equally.
[†]These authors jointly supervised.

Supplementary Table 1: Details of LNP libraries sourced for LNPDB. LNPDB compiles diverse structural and functional data for 19,528 LNP formulations, representing 12,845 unique ionizable lipids across 42 publications and one commercial supplier, BroadPharm.

Library	# LNPs	# unique ionizable lipids	Cargo	Delivery target	Reference
AA_2008	497	487	siRNA	in vitro	https://www.nature.com/articles/nbt1402
KL_2010	378	378	siRNA	in vitro	https://www.pnas.org/doi/full/10.1073/pnas.0910603106
LL_2012	112	112	pDNA	in vitro	https://www.sciencedirect.com/science/article/pii/S0142961212008393
KW_2014	1273	1158	siRNA	in vitro, liver	https://www.nature.com/articles/ncomms5277
JM_2016	72	72	siRNA	in vitro	https://onlinelibrary.wiley.com/doi/10.1002/anie.201610209
KZ_2016	1538	1470	siRNA	in vitro, liver	https://www.pnas.org/doi/10.1073/pnas.1520756113
SS_2018	10	10	mRNA	whole body	https://doi.org/10.1016/j.ymthe.2018.03.010
LM_2019	1128	1080	mRNA	in vitro	https://www.nature.com/articles/s41587-019-0247-3
SL_2020	91	84	mRNA	in vitro	https://onlinelibrary.wiley.com/doi/10.1002/anie.202013927
SP_2020	30	1	mRNA	in vitro	https://www.nature.com/articles/s41467-020-14527-2
SL_2021	572	572	mRNA	in vitro	https://www.nature.com/articles/s41563-020-00886-0
YZ_2022	1080	1	pDNA	in vitro	https://www.nature.com/articles/s41467-022-31993-y
ZL_2022	144	144	mRNA	in vitro	https://pubs.acs.org/doi/10.1021/acsnano.2c07822
BL_2023	773	711	mRNA	in vitro, muscle, lung epithelium	https://www.nature.com/articles/s41587-023-01679-x
JC_2023	288	288	mRNA	in vitro	https://www.pnas.org/doi/10.1073/pnas.2309472120
LR_2023	407	384	mRNA	liver	https://www.nature.com/articles/s41551-023-01030-4
RG_2023	260	260	mRNA	muscle	https://doi.org/10.1016/j.biomaterials.2023.122243
YX_2023	248	176	mRNA	in vitro	https://onlinelibrary.wiley.com/doi/10.1002/adhm.202302691
YY_2023	119	119	mRNA	in vitro	https://doi.org/10.1016/j.biomaterials.2023.122279
ZC_2023	131	101	mRNA	in vitro, liver	https://pubs.acs.org/doi/full/10.1021/jacs.3c09143
ZH_2023	325	161	mRNA	liver, spleen	https://onlinelibrary.wiley.com/doi/10.1002/anie.202310401
BL_2024	611	560	mRNA	in vitro, muscle	https://www.nature.com/articles/s41563-024-01867-3
BroadPharm_2024		269			https://broadpharm.com/product-categories/lipid/ionizable-lipid
JL_2024	623	623	mRNA	in vitro	https://jnanobiotechnology.biomedcentral.com/articles/10.1186/s12951-024-02919-1
JW_2024	1902	265	mRNA	muscle, lung epithelium	https://www.nature.com/articles/s41587-024-02490-y
KS_2024	140	140	mRNA	in vitro	https://www.nature.com/articles/s41467-024-50093-7
LX_2024	851	180	mRNA	in vitro, heart, lung, kidney, spleen, liver, multiorgan	https://www.nature.com/articles/s41467-024-45422-9
LX_2024_2	270	270	mRNA	in vitro	https://pubs.acs.org/doi/10.1021/jacs.4c10265
LX_2024_3	252	252	mRNA	in vitro	https://www.nature.com/articles/s41565-024-01747-6
NC_2024	350	260	mRNA	in vitro	https://www.pnas.org/doi/10.1073/pnas.2307810121

SB_2024	24	2	mRNA	ear	https://doi.org/10.1016/j.bioactmat.2024.05.012
SW_2024	180	180	mRNA	in vitro	https://www.sciencedirect.com/science/article/abs/pii/S0168365924007016
XH_2024	60	30	mRNA	in vitro, liver	https://www.nature.com/articles/s41467-024-45537-z
XH_2024_2	151	151	mRNA	in vitro, liver	https://www.nature.com/articles/s41551-024-01267-7
YR_2024	286	286	mRNA	in vitro	https://pubs.rsc.org/en/Content/ArticleLanding/2024/TB/D4TB00960F
YX_2024	2400	1100	mRNA	in vitro	https://www.nature.com/articles/s41467-024-50619-z
YZ_2024	1080	1	mRNA	in vitro	https://www.nature.com/articles/s41551-023-01131-0
AP_2025	91	91	mRNA	muscle	https://www.nature.com/articles/s42004-025-01516-z
LL_2025	8	1	mRNA	muscle	https://doi.org/10.1039/D5NR00433K
LZ_2025	96	1	mRNA	in vitro	https://doi.org/10.1016/j.jconrel.2025.01.071
SX_2025	141	130	mRNA	bone marrow	https://www.nature.com/articles/s41551-025-01480-y
SY_2025	35	18	mRNA	ear	https://doi.org/10.1002/adhm.202403366
XH_2025	500	500	mRNA	in vitro	https://www.biorxiv.org/content/10.1101/2025.02.25.640222v1

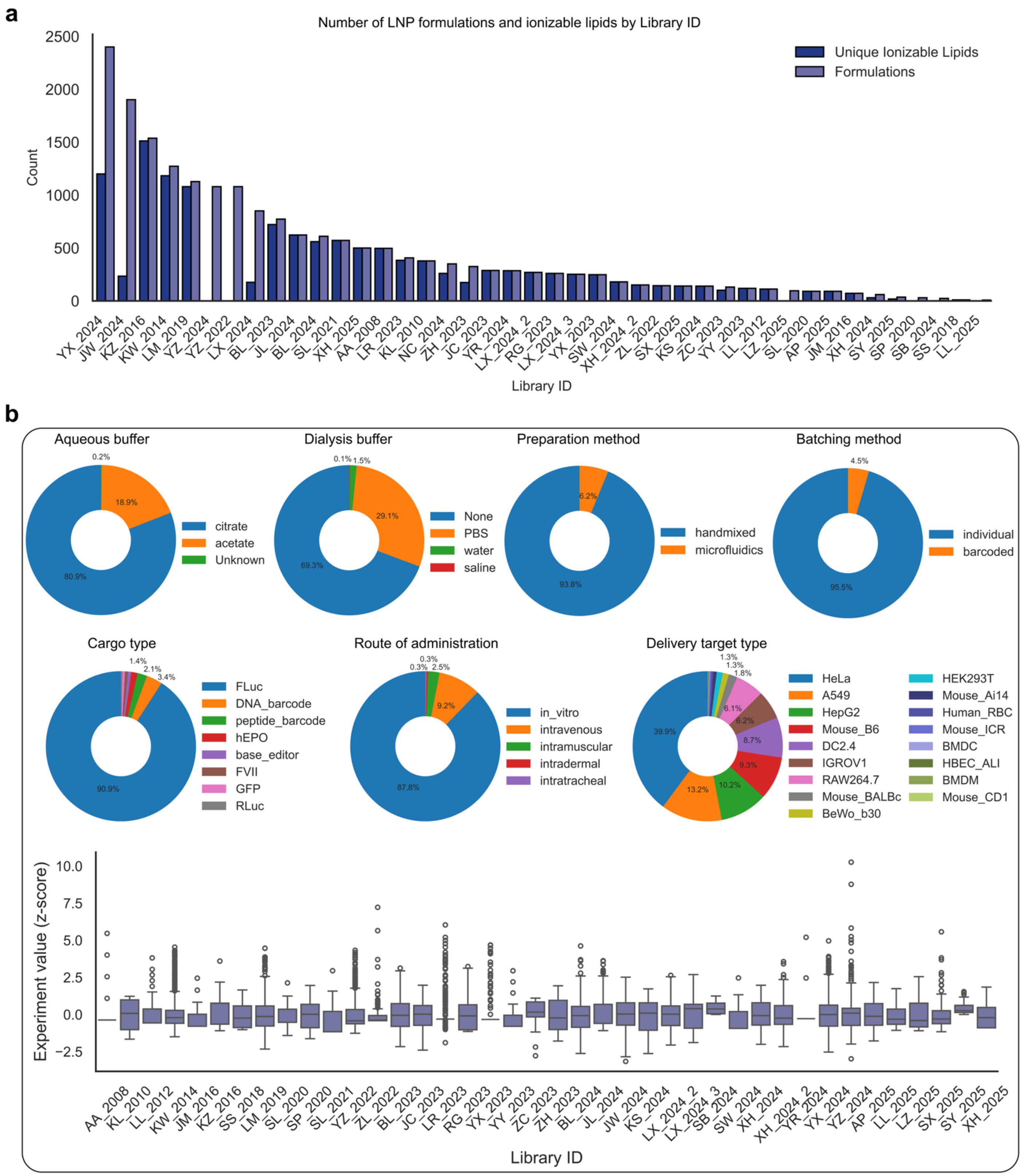

Supplementary Fig. 1: Additional summary statistics for LNP data in LNPDB. a Number of LNP formulations and unique ionizable lipids for each of the 42 libraries available in LNPDB. There are 269 additional unique ionizable lipids from BroadPharm (not shown) available in LNPDB. **b** Additional summary statistics of properties by aqueous buffer, dialysis buffer, preparation method, batching method, cargo type, route of administration, and delivery target type. Distributions of experiment value *z*-scores for each library are plotted. Experiment values not denoting delivery

performance were omitted from this analysis. All boxplots have a box that signifies the interquartile range (*IQR*; 25th percentile to 75th percentile), a center bar that denotes the median, whiskers that extend up to 1.5 × *IQR*, and a notch that extends 1.58 × $IQR/\sqrt{n}$, where *n* is the sample size for that condition, to estimate the 95% confidence interval. Source data are provided as a Source data file.

Supplementary Table 2: Details of bilayer assembly simulations referenced in this study. All simulations were executed using OpenMM. Initial bilayers were assembled using Membrane Builder in MolCube-Builder. Formulations whose bilayers were consistently stable (i.e., preserved intact bilayer) were simulated for 1.5 μs. Formulations whose bilayers were not stable (e.g., ionizable lipids escaped bilayer) were stopped before reaching 1.5 μs. White rows denote systems with 50% protonated, 50% neutral ionizable lipids; gray rows denote systems with entirely neutral ionizable lipids.

ID	LNP ID	IL name	HL name	# molecules per leaflet					water thickness	XYZ (Å^3)	# Na	# Cl	# waters	# atoms	Duration (ns)	Temp (K)
				IL (P)	IL (N)	HL	Chol	PEG								
LM_1	LNP_0003881	LM_A12Iso52DC18	DOPE	18	18	16	47	0	20	84x84x85	24	60	8876	47552	1,500	310
LM_2	LNP_0003882	LM_A2Iso52DC18	DOPE	18	18	16	47	0	20	85x85x91	28	64	10204	52248	1,500	310
LM_3	LNP_0003883	LM_A3Iso52DC18	DOPE	18	18	16	47	0	20	84x84x85	24	60	8795	47957	1,500	310
LM_4	LNP_0003898	LM_A2Iso92DC18	DOPE	18	18	16	47	0	20	83x83x91	26	62	9488	50816	1,500	310
LM_5	LNP_0003901	LM_A12Iso92DC18	DOPE	18	18	16	47	0	20	83x83x90	26	62	9416	49896	1,500	310
LM_6	LNP_0003904	LM_A3Iso92DC18	DOPE	18	18	16	47	0	20	84x84x86	24	60	8934	48878	1,500	310
LM_7	LNP_0004015	LM_A2Iso5C11	DOPE	18	18	16	47	0	20	84x84x86	24	60	8891	45149	1,500	310
LM_8	LNP_0004019	LM_A2Iso9C11	DOPE	18	18	16	47	0	20	83x83x89	26	62	9251	46953	1,500	310
LM_9	LNP_0004025	LM_A2Iso5C10	DOPE	18	18	16	47	0	20	82x82x91	26	62	9370	46574	1,500	310
LM_10	LNP_0004029	LM_A2Iso9C10	DOPE	18	18	16	47	0	20	84x84x100	28	64	10485	50643	1,500	310
LM_11	LNP_0004035	LM_A2Iso5C9	DOPE	18	18	16	47	0	20	84x84x85	24	60	8872	44228	1,500	310
LM_12	LNP_0004039	LM_A2Iso9C9	DOPE	18	18	16	47	0	20	84x84x92	26	62	9576	47480	1,500	310
LM_13	LNP_0004045	LM_A2Iso5C8	DOPE	18	18	16	47	0	20	84x84x86	24	60	8921	43943	100	310
LM_14	LNP_0004049	LM_A2Iso9C8	DOPE	18	18	16	47	0	20	87x87x91	28	64	10310	49254	1,500	310
LM_15	LNP_0004055	LM_A2Iso5C7	DOPE	18	18	16	47	0	20	84x84x91	28	64	9976	47100	100	310
LM_16	LNP_0004059	LM_A2Iso9C7	DOPE	18	18	16	47	0	20	85x85x92	28	64	9832	47388	300	310
LM_17	LNP_0004065	LM_A2Iso5CC7	DOPE	18	18	16	47	0	20	85x85x87	26	62	9426	45318	100	310
LM_18	LNP_0004069	LM_A2Iso9CC7	DOPE	18	18	16	47	0	20	84x84x89	26	62	9410	45990	200	310
LM_19	LNP_0004075	LM_A2Iso5CC6	DOPE	18	18	16	47	0	20	84x84x86	24	60	8870	42998	100	310
LM_20	LNP_0004079	LM_A2Iso9CC6	DOPE	18	18	16	47	0	20	83x83x105	30	66	11145	50555	100	310
LM_21	LNP_0004085	LM_A2Iso5C6	DOPE	18	18	16	47	0	20	84x84x85	24	60	8856	42884	100	310
LM_22	LNP_0004089	LM_A2Iso9C6	DOPE	18	18	16	47	0	20	85x85x88	26	62	9499	45537	100	310
LM_23	LNP_0004102	LM_A3Iso5C11	DOPE	18	18	16	47	0	20	85x85x85	26	62	9050	45990	1,500	310
LM_24	LNP_0004106	LM_A3Iso9C11	DOPE	18	18	16	47	0	20	83x83x86	24	60	8582	45086	1,500	310
LM_25	LNP_0004112	LM_A3Iso5C10	DOPE	18	18	16	47	0	20	84x84x86	24	60	8944	45236	1,500	310
LM_26	LNP_0004116	LM_A3Iso9C10	DOPE	18	18	16	47	0	20	86x86x85	26	62	9203	46521	1,500	310
LM_27	LNP_0004122	LM_A3Iso5C9	DOPE	18	18	16	47	0	20	83x83x86	24	60	8803	44381	1,500	310
LM_28	LNP_0004126	LM_A3Iso9C9	DOPE	18	18	16	47	0	20	83x83x87	24	60	8874	45098	1,500	310
LM_29	LNP_0004132	LM_A3Iso5C8	DOPE	18	18	16	47	0	20	83x83x87	24	60	8964	44432	1,500	310
LM_30	LNP_0004136	LM_A3Iso9C8	DOPE	18	18	16	47	0	20	84x84x86	24	60	8944	44876	1,500	310
LM_31	LNP_0004142	LM_A3Iso5C7	DOPE	18	18	16	47	0	20	85x85x85	24	60	8965	44003	1,500	310
LM_32	LNP_0004146	LM_A3Iso9C7	DOPE	18	18	16	47	0	20	83x83x88	26	62	9053	44775	1,500	310
LM_33	LNP_0004152	LM_A3Iso5CC7	DOPE	18	18	16	47	0	20	82x82x84	24	60	8356	42464	1,500	310
LM_34	LNP_0004156	LM_A3Iso9CC7	DOPE	18	18	16	47	0	20	83x83x87	24	60	8871	44513	1,500	310
LM_35	LNP_0004162	LM_A3Iso5CC6	DOPE	18	18	16	47	0	20	83x83x86	24	60	8918	43502	1,500	310
LM_36	LNP_0004166	LM_A3Iso9CC6	DOPE	18	18	16	47	0	20	83x83x85	24	60	8662	43238	200	310

LM_37	LNP_0004172	LM_A3Iso5C6	DOPE	18	18	16	47	0	20	85x85x85	26	62	9076	43908	400	310
LM_38	LNP_0004176	LM_A3Iso9C6	DOPE	18	18	16	47	0	20	83x83x86	24	60	8814	43622	700	310
LM_39	LNP_0004885	LM_A12Iso5C11	DOPE	18	18	16	47	0	20	83x83x84	24	60	8587	43949	1,500	310
LM_40	LNP_0004889	LM_A12Iso9C11	DOPE	18	18	16	47	0	20	83x83x85	24	60	8562	44594	1,500	310
LM_41	LNP_0004895	LM_A12Iso5C10	DOPE	18	18	16	47	0	20	85x85x85	26	62	9037	44871	1,500	310
LM_42	LNP_0004899	LM_A12Iso9C10	DOPE	18	18	16	47	0	20	83x83x85	24	60	8611	44309	1,500	310
LM_43	LNP_0004905	LM_A12Iso5C9	DOPE	18	18	16	47	0	20	85x85x85	24	60	9023	44393	1,500	310
LM_44	LNP_0004909	LM_A12Iso9C9	DOPE	18	18	16	47	0	20	84x84x85	24	60	8847	44585	1,500	310
LM_45	LNP_0004915	LM_A12Iso5C8	DOPE	18	18	16	47	0	20	84x84x85	24	60	8849	43439	1,500	310
LM_46	LNP_0004919	LM_A12Iso9C8	DOPE	18	18	16	47	0	20	84x84x85	24	60	8858	44186	1,500	310
LM_47	LNP_0004925	LM_A12Iso5C7	DOPE	18	18	16	47	0	20	84x84x85	24	60	8818	42914	1,500	310
LM_48	LNP_0004929	LM_A12Iso9C7	DOPE	18	18	16	47	0	20	83x83x85	24	60	8592	42956	1,500	310
LM_49	LNP_0004935	LM_A12Iso5CC7	DOPE	18	18	16	47	0	20	85x85x85	24	60	9041	43871	1,500	310
LM_50	LNP_0004939	LM_A12Iso9CC7	DOPE	18	18	16	47	0	20	82x82x85	24	60	8364	42560	1,500	310
LM_51	LNP_0004945	LM_A12Iso5CC6	DOPE	18	18	16	47	0	20	83x83x85	24	60	8680	42140	1,500	310
LM_52	LNP_0004949	LM_A12Iso9CC6	DOPE	18	18	16	47	0	20	82x82x86	24	60	8430	42110	1,500	310
LM_53	LNP_0004955	LM_A12Iso5C6	DOPE	18	18	16	47	0	20	83x83x84	24	60	8542	41654	1,500	310
LM_54	LNP_0004959	LM_A12Iso9C6	DOPE	18	18	16	47	0	20	84x84x86	24	60	8909	43475	480	310
LM_1	LNP_0003881	LM_A12Iso52DC18	DOPE	0	35	16	47	0	10	83x83x66	12	12	4513	34367	1,500	310
LM_2	LNP_0003882	LM_A2Iso52DC18	DOPE	0	35	16	47	0	10	84x84x65	12	12	4514	34380	1,500	310
LM_3	LNP_0003883	LM_A3Iso52DC18	DOPE	0	35	16	47	0	10	85x85x67	14	14	4881	35835	1,500	310
LM_4	LNP_0003898	LM_A2Iso92DC18	DOPE	0	35	16	47	0	10	83x83x70	14	14	5008	36566	1,500	310
LM_5	LNP_0003901	LM_A12Iso92DC18	DOPE	0	35	16	47	0	10	87x87x67	14	14	5295	37437	1,500	310
LM_6	LNP_0003904	LM_A3Iso92DC18	DOPE	0	35	16	47	0	10	83x83x69	14	14	5007	36703	1,500	310
LM_7	LNP_0004015	LM_A2Iso5C11	DOPE	0	35	16	47	0	10	85x85x66	12	12	4627	32059	1,500	310
LM_8	LNP_0004019	LM_A2Iso9C11	DOPE	0	35	16	47	0	10	84x84x67	12	12	4737	33089	1,500	310
LM_9	LNP_0004025	LM_A2Iso5C10	DOPE	0	35	16	47	0	10	83x83x65	12	12	4374	30880	1,500	310
LM_10	LNP_0004029	LM_A2Iso9C10	DOPE	0	35	16	47	0	10	85x85x80	18	18	6638	38384	1,500	310
LM_11	LNP_0004035	LM_A2Iso5C9	DOPE	0	35	16	47	0	10	82x82x66	12	12	4306	30256	1,500	310
LM_12	LNP_0004039	LM_A2Iso9C9	DOPE	0	35	16	47	0	10	83x83x65	12	12	4279	30875	1,500	310
LM_13	LNP_0004045	LM_A2Iso5C8	DOPE	0	35	16	47	0	10	84x84x65	12	12	4306	29836	1,500	310
LM_14	LNP_0004049	LM_A2Iso9C8	DOPE	0	35	16	47	0	10	84x84x66	12	12	4470	31028	1,500	310
LM_15	LNP_0004055	LM_A2Iso5C7	DOPE	0	35	16	47	0	10	84x84x65	12	12	4396	29686	1,500	310
LM_16	LNP_0004059	LM_A2Iso9C7	DOPE	0	35	16	47	0	10	81x81x65	12	12	4103	29507	1,500	310
LM_17	LNP_0004065	LM_A2Iso5CC7	DOPE	0	35	16	47	0	10	83x83x66	12	12	4368	29882	1,500	310
LM_18	LNP_0004069	LM_A2Iso9CC7	DOPE	0	35	16	47	0	10	83x83x80	18	18	6563	37179	1,500	310
LM_19	LNP_0004075	LM_A2Iso5CC6	DOPE	0	35	16	47	0	10	85x85x66	12	12	4568	29852	600	310
LM_20	LNP_0004079	LM_A2Iso9CC6	DOPE	0	35	16	47	0	10	82x82x67	12	12	4441	30171	1,500	310
LM_21	LNP_0004085	LM_A2Iso5C6	DOPE	0	35	16	47	0	10	85x85x66	12	12	4705	30193	600	310
LM_22	LNP_0004089	LM_A2Iso9C6	DOPE	0	35	16	47	0	10	84x84x66	12	12	4541	30401	1,500	310
LM_23	LNP_0004102	LM_A3Iso5C11	DOPE	0	35	16	47	0	10	83x83x65	12	12	4473	31947	1,500	310
LM_24	LNP_0004106	LM_A3Iso9C11	DOPE	0	35	16	47	0	10	83x83x64	12	12	4156	31486	1,500	310
LM_25	LNP_0004112	LM_A3Iso5C10	DOPE	0	35	16	47	0	10	83x83x64	12	12	4316	31056	1,500	310

LM_26	LNP_0004116	LM_A3Iso9C10	DOPE	0	35	16	47	0	10	82x82x63	10	10	3865	30189	1,500	310
LM_27	LNP_0004122	LM_A3Iso5C9	DOPE	0	35	16	47	0	10	83x83x64	12	12	4287	30549	1,500	310
LM_28	LNP_0004126	LM_A3Iso9C9	DOPE	0	35	16	47	0	10	83x83x65	12	12	4305	31093	1,500	310
LM_29	LNP_0004132	LM_A3Iso5C8	DOPE	0	35	16	47	0	10	82x82x65	12	12	4305	30183	1,500	310
LM_30	LNP_0004136	LM_A3Iso9C8	DOPE	0	35	16	47	0	10	82x82x65	12	12	4256	30526	1,500	310
LM_31	LNP_0004142	LM_A3Iso5C7	DOPE	0	35	16	47	0	10	85x85x65	12	12	4742	31074	1,500	310
LM_32	LNP_0004146	LM_A3Iso9C7	DOPE	0	35	16	47	0	10	83x83x64	12	12	4275	30163	1,500	310
LM_33	LNP_0004152	LM_A3Iso5CC7	DOPE	0	35	16	47	0	10	84x84x65	12	12	4482	30574	1,500	310
LM_34	LNP_0004156	LM_A3Iso9CC7	DOPE	0	35	16	47	0	10	84x84x65	12	12	4626	31496	1,500	310
LM_35	LNP_0004162	LM_A3Iso5CC6	DOPE	0	35	16	47	0	10	82x82x67	12	12	4597	30289	1,500	310
LM_36	LNP_0004166	LM_A3Iso9CC6	DOPE	0	35	16	47	0	10	82x82x65	12	12	4245	29723	1,500	310
LM_37	LNP_0004172	LM_A3Iso5C6	DOPE	0	35	16	47	0	10	82x82x65	12	12	4257	29199	1,500	310
LM_38	LNP_0004176	LM_A3Iso9C6	DOPE	0	35	16	47	0	10	84x84x65	12	12	4586	30676	1,500	310
LM_39	LNP_0004885	LM_A12Iso5C11	DOPE	0	35	16	47	0	10	84x84x65	12	12	4556	31760	1,500	310
LM_40	LNP_0004889	LM_A12Iso9C11	DOPE	0	35	16	47	0	10	83x83x65	12	12	4368	31916	1,500	310
LM_41	LNP_0004895	LM_A12Iso5C10	DOPE	0	35	16	47	0	10	85x85x66	12	12	4765	31955	1,500	310
LM_42	LNP_0004899	LM_A12Iso9C10	DOPE	0	35	16	47	0	10	84x84x66	12	12	4634	32282	1,500	310
LM_43	LNP_0004905	LM_A12Iso5C9	DOPE	0	35	16	47	0	10	82x82x65	12	12	4372	30344	1,500	310
LM_44	LNP_0004909	LM_A12Iso9C9	DOPE	0	35	16	47	0	10	83x83x64	12	12	4207	30569	1,500	310
LM_45	LNP_0004915	LM_A12Iso5C8	DOPE	0	35	16	47	0	10	83x83x65	12	12	4538	30410	1,500	310
LM_46	LNP_0004919	LM_A12Iso9C8	DOPE	0	35	16	47	0	10	83x83x65	12	12	4370	30626	1,500	310
LM_47	LNP_0004925	LM_A12Iso5C7	DOPE	0	35	16	47	0	10	82x82x65	12	12	4346	29402	1,500	310
LM_48	LNP_0004929	LM_A12Iso9C7	DOPE	0	35	16	47	0	10	84x84x65	12	12	4360	30164	1,500	310
LM_49	LNP_0004935	LM_A12Iso5CC7	DOPE	0	35	16	47	0	10	84x84x66	12	12	4657	30623	1,500	310
LM_50	LNP_0004939	LM_A12Iso9CC7	DOPE	0	35	16	47	0	10	84x84x64	12	12	4393	30551	1,500	310
LM_51	LNP_0004945	LM_A12Iso5CC6	DOPE	0	35	16	47	0	10	84x84x65	12	12	4580	29744	1,500	310
LM_52	LNP_0004949	LM_A12Iso9CC6	DOPE	0	35	16	47	0	10	83x83x65	12	12	4332	29720	1,500	310
LM_53	LNP_0004955	LM_A12Iso5C6	DOPE	0	35	16	47	0	10	83x83x65	12	12	4398	29126	1,500	310
LM_54	LNP_0004959	LM_A12Iso9C6	DOPE	0	35	16	47	0	10	87x87x64	12	12	4594	30434	1,500	310
EC_13		SM-102	DSPC	25	25	10	39	0	20	88x88x90	30	80	10698	54566	1,500	310
EC_23	LNP_0009476	DLin-MC3-DMA	DSPC	25	25	10	39	0	20	87x87x90	30	80	10749	53519	1,500	310
EC_27		ALC-0315	DOPE	18	18	16	47	0	20	83x83x92	28	64	9743	51585	1,500	310
EC_13		SM-102	DSPC	0	50	10	39	0	10	87x87x71	16	18	5947	40185	1,500	310
EC_23	LNP_0009476	DLin-MC3-DMA	DSPC	0	50	10	39	0	10	87x87x71	18	18	6445	40483	1,500	310
EC_27		ALC-0315	DOPE	0	35	16	47	0	10	85x85x63	12	12	4355	34603	1,500	310
LM_1	LNP_0003881	LM_A12Iso52DC18	DOPE	0	35	16	47	0	10	83x83x66	12	12	4508	34082	1,500	298
LM_5	LNP_0003901	LM_A12Iso92DC18	DOPE	0	35	16	47	0	10	86x86x67	14	14	4794	35644	1,500	298
LM_39	LNP_0004885	LM_A12Iso5C11	DOPE	0	35	16	47	0	10	83x83x66	12	12	4486	31356	1,500	298
LM_40	LNP_0004889	LM_A12Iso9C11	DOPE	0	35	16	47	0	10	84x84x64	12	12	4310	31528	1,500	298
LM_41	LNP_0004895	LM_A12Iso5C10	DOPE	0	35	16	47	0	10	82x82x64	12	12	4111	29811	1,500	298
LM_42	LNP_0004899	LM_A12Iso9C10	DOPE	0	35	16	47	0	10	81x81x65	12	12	4276	31006	1,500	298
LM_44	LNP_0004909	LM_A12Iso9C9	DOPE	0	35	16	47	0	10	83x83x65	12	12	4317	30709	1,500	298
LM_46	LNP_0004919	LM_A12Iso9C8	DOPE	0	35	16	47	0	10	82x82x65	12	12	4386	30496	1,500	298

LM_48	LNP_0004929	LM_A12Iso9C7	DOPE	0	35	16	47	0	10	83x83x64	12	12	4178	29452	1,500	298
LM_50	LNP_0004939	LM_A12Iso9CC7	DOPE	0	35	16	47	0	10	84x84x63	12	12	4530	30788	1,500	298
LM_1	LNP_0003881	LM_A12Iso52DC18	DOPE	0	35	16	47	2	15	82x82x126	46	46	16849	72269	1,500	310
LM_5	LNP_0003901	LM_A12Iso92DC18	DOPE	0	35	16	47	2	15	83x83x123	46	46	16500	71922	1,500	310
LM_39	LNP_0004885	LM_A12Iso5C11	DOPE	0	35	16	47	2	15	85x85x129	52	52	18825	75549	1,500	310
LM_40	LNP_0004889	LM_A12Iso9C11	DOPE	0	35	16	47	2	15	84x84x112	40	40	14791	64123	1,500	310
LM_41	LNP_0004895	LM_A12Iso5C10	DOPE	0	35	16	47	2	15	84x84x122	46	46	16782	68988	1,500	310
LM_42	LNP_0004899	LM_A12Iso9C10	DOPE	0	35	16	47	2	15	85x85x114	42	42	15548	65978	1,500	310
LM_44	LNP_0004909	LM_A12Iso9C9	DOPE	0	35	16	47	2	15	84x84x141	58	58	20953	81805	1,500	310
LM_46	LNP_0004919	LM_A12Iso9C8	DOPE	0	35	16	47	2	15	83x83x125	48	48	17140	69926	1,500	310
LM_48	LNP_0004929	LM_A12Iso9C7	DOPE	0	35	16	47	2	15	81x81x119	42	42	14941	62897	1,500	310
LM_50	LNP_0004939	LM_A12Iso9CC7	DOPE	0	35	16	47	2	15	84x84x114	42	42	15257	64125	1,500	310

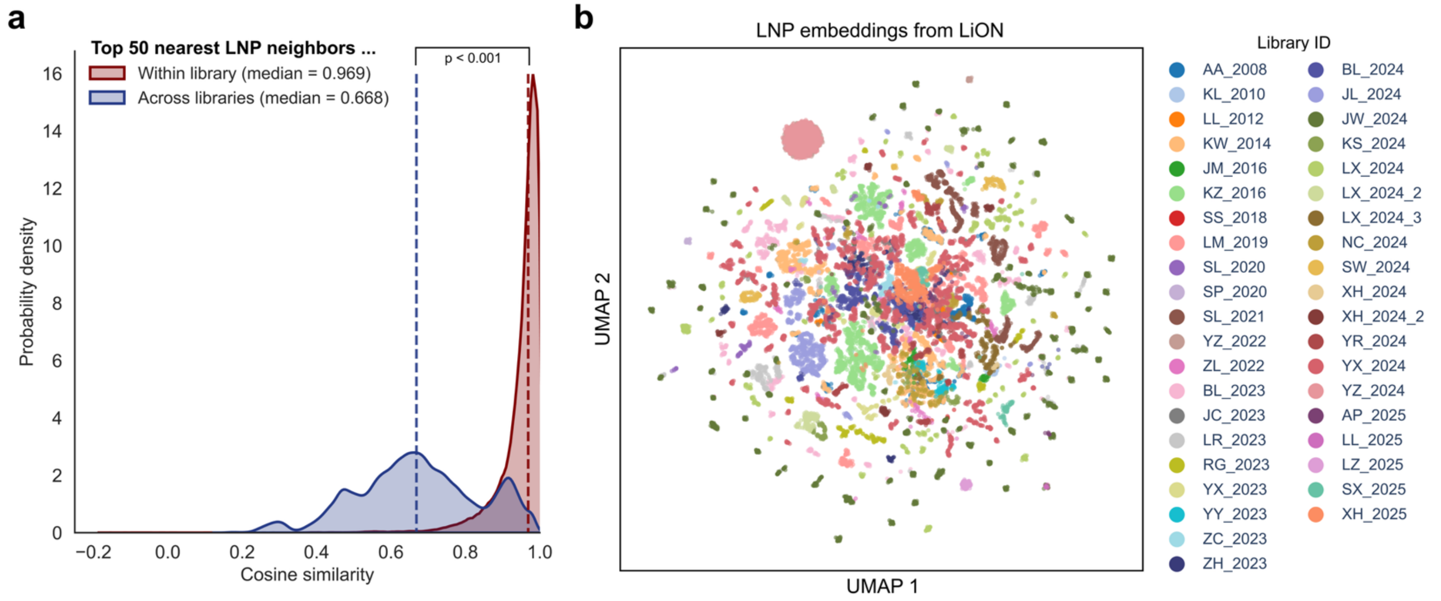

Supplementary Fig. 2: Additional LNP embedding analysis. a Probability distributions of cosine similarities for the top 50 nearest neighbors for each LNP, computed separately within library and across libraries. This analysis is based on the original LNP embedding space shown in Fig. 2a prior to UMAP dimensionality reduction. Probability density functions are plotted, with vertical lines indicating median values and significant Kolmogorov-Smirnov *p* value shown (Kolmogorov-Smirnov statistic = 0.76). Intra-library LNP neighbors show higher similarity, reflecting greater homogeneity within studies, whereas inter-experiment LNP neighbors are more broadly distributed. **b** UMAP visualization of high-dimensional embedding landscape (i.e., fingerprints) from LiON model of LNP formulations. Fingerprints were extracted from the penultimate linear layer of the LiON model's feedforward neural network trained on LNPDB as shown in Fig. 3a. This is an alternative embedding approach to that visualized in Fig. 2a. Source data are provided as a Source data file.

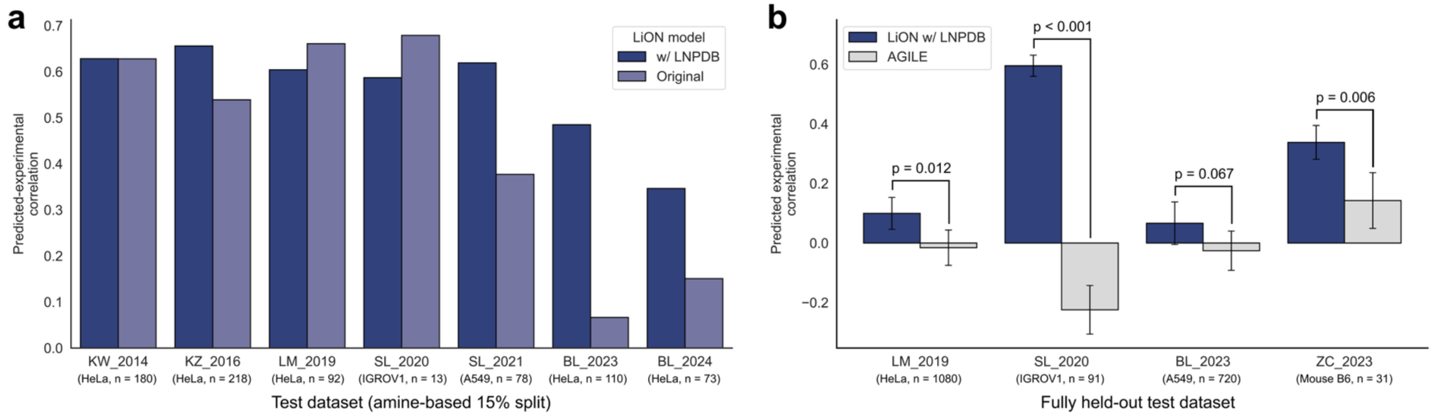

Supplementary Fig. 3: Pearson correlation results for deep learning models. a Analogous to Fig. 3a but measured using Pearson correlation. **b** Analogous to Fig. 3b but measured using Pearson correlation. Source data are provided as a Source data file.

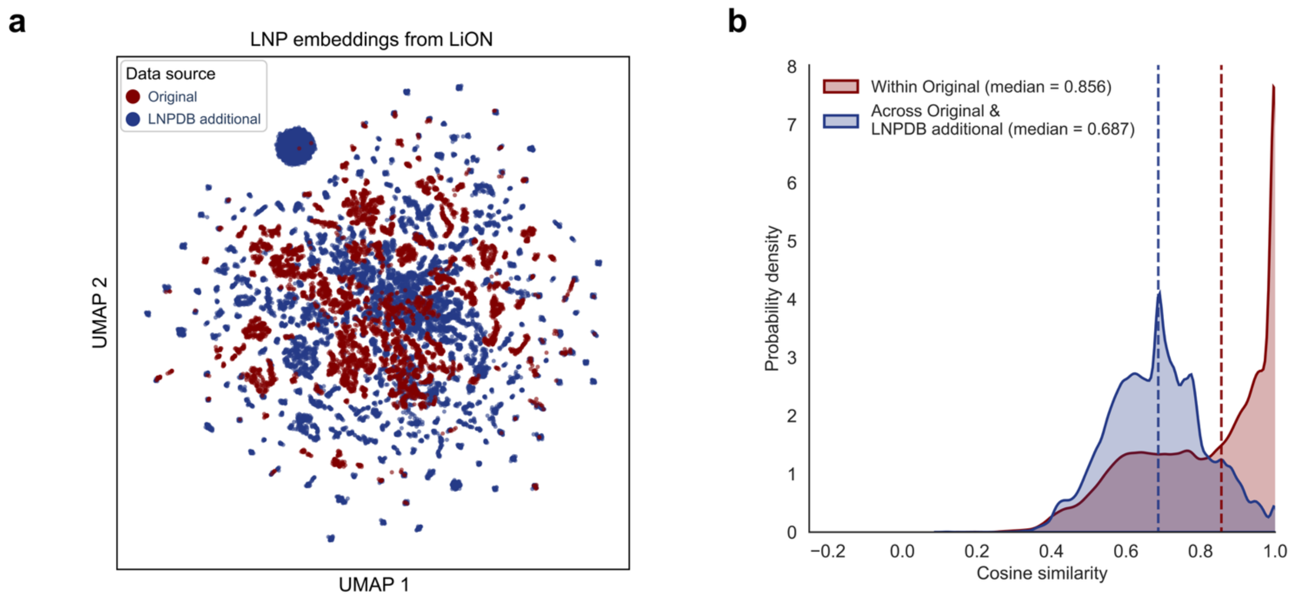

Supplementary Fig. 4: Expanded data of LNPDB blends with original data in LNP embedding space learned by LiON. a Analogous to Supplementary Fig. 2b but colored by whether the training data were sourced from the original dataset ("Original") or data newly included in LNPDB ("LNPDB additional"). Note that LNPDB altogether contains the data of both "Original" and "LNPDB additional". The LiON fingerprints of these two groups are interspersed, suggesting shared structure–function patterns and that the additional data densifies the representation space. **b** Analogous to Supplementary Fig. 2a, displaying probability distributions of cosine similarities in LiON embedding space for the top 50 nearest neighbors for each LNP, computed separately within the original dataset and across the original dataset and data newly included in LNPDB. The presence of some high pairwise similarities across the original dataset and the data newly included in LNPDB similarly reinforce the suitability of using the combined dataset to learn shared structure-function relationships. Moreover, LNPs in the expanded dataset that are relatively dissimilar to those of the original dataset broaden the chemical design space, potentially enabling exploration and learning in under-sampled regions. Source data are provided as a Source data file.

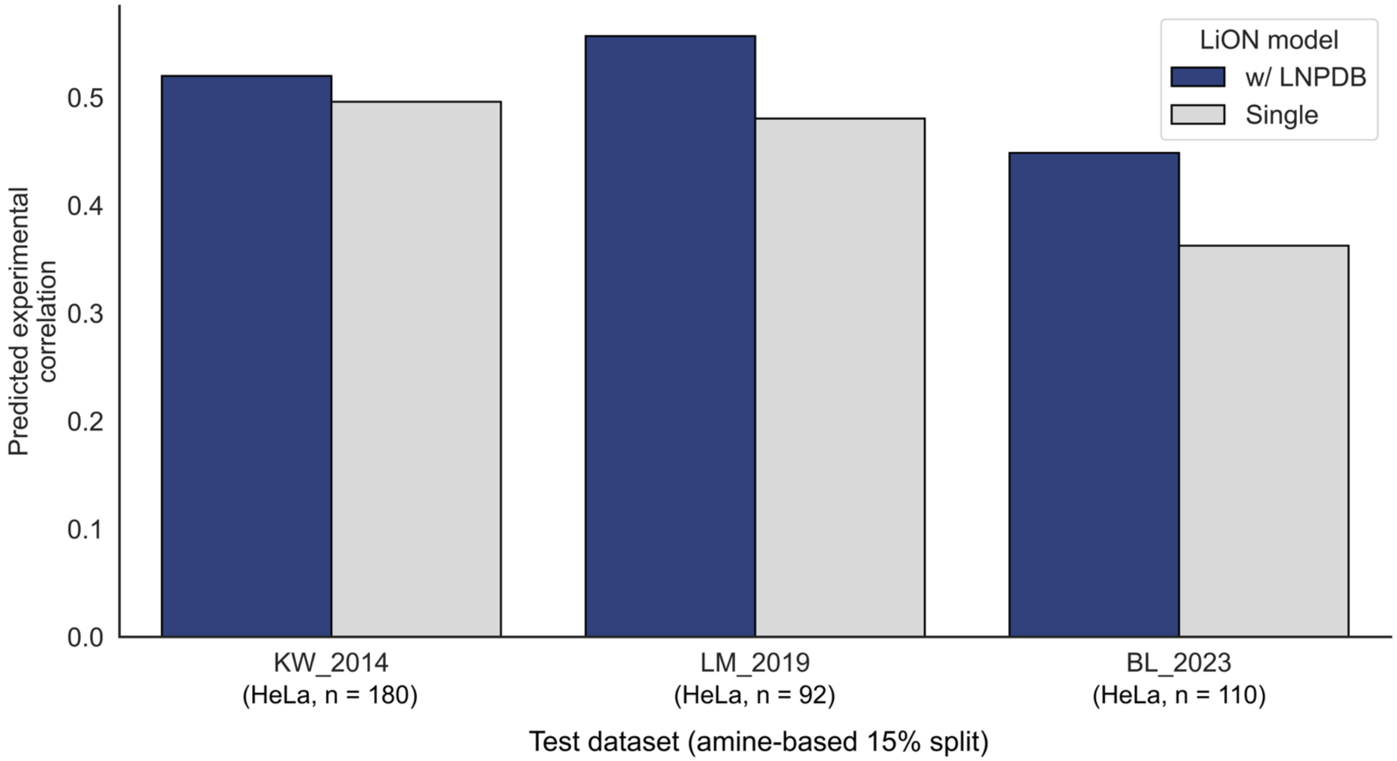

Supplementary Fig. 5: Learning from outside datasets improves predictive performance of LiON. Training LiON exclusively on a single dataset yields poorer predictive performance (measured by Spearman correlation) than when trained on LNPDB. This suggests that integrating data from multiple studies enables the model to capture broader structure-function relationships. Source data are provided as a Source data file.

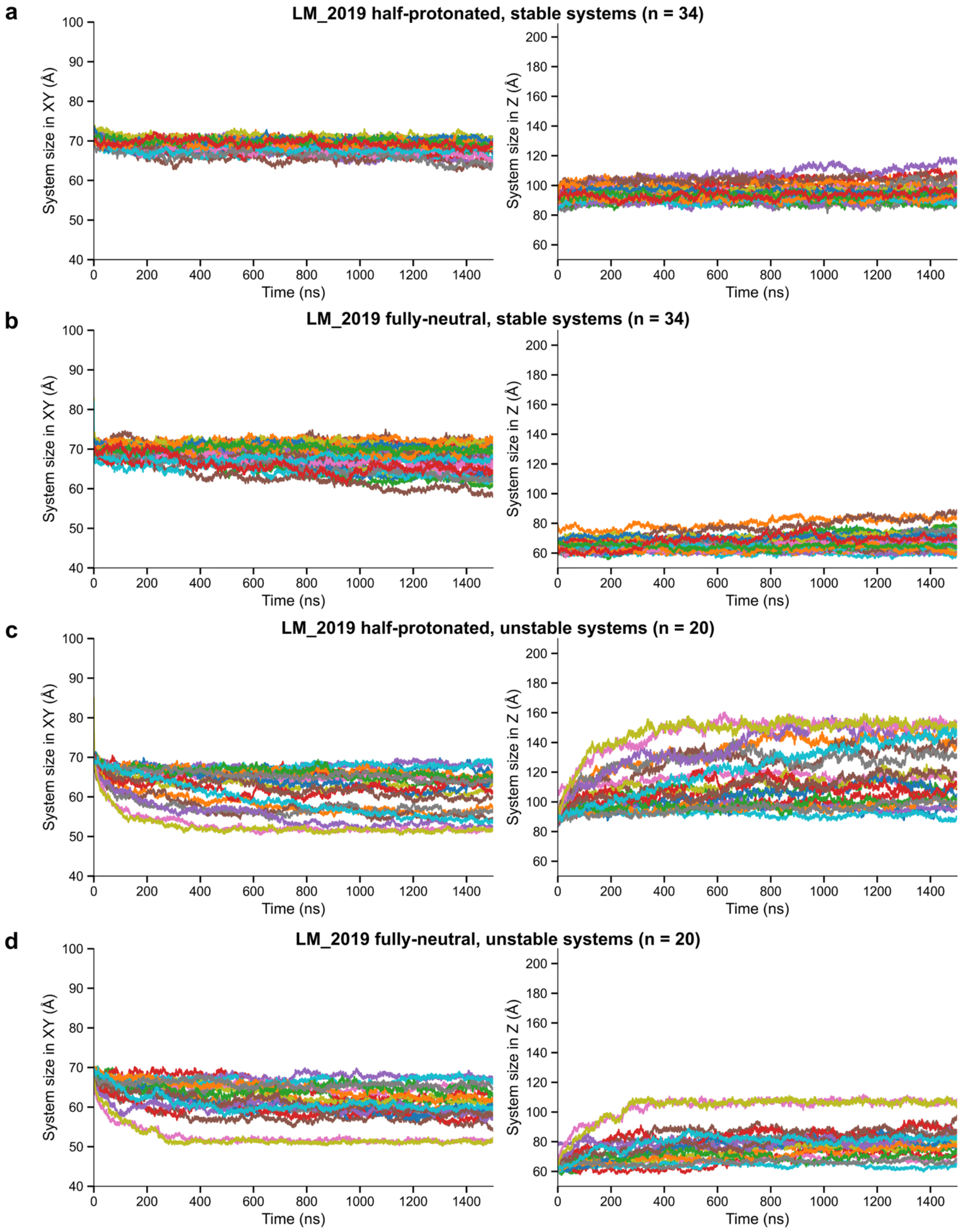

Supplementary Fig. 6: System size for select LM_2019 LNP bilayers over the course of simulations. All trajectories extend to 1.5 µs and correspond to LM_2019[1] bilayers assessed in this study. Executed with OpenMM with 100 lipids per leaflet. Trajectories grouped based on protonation and stability conditions. Source data are provided as a Source data file.

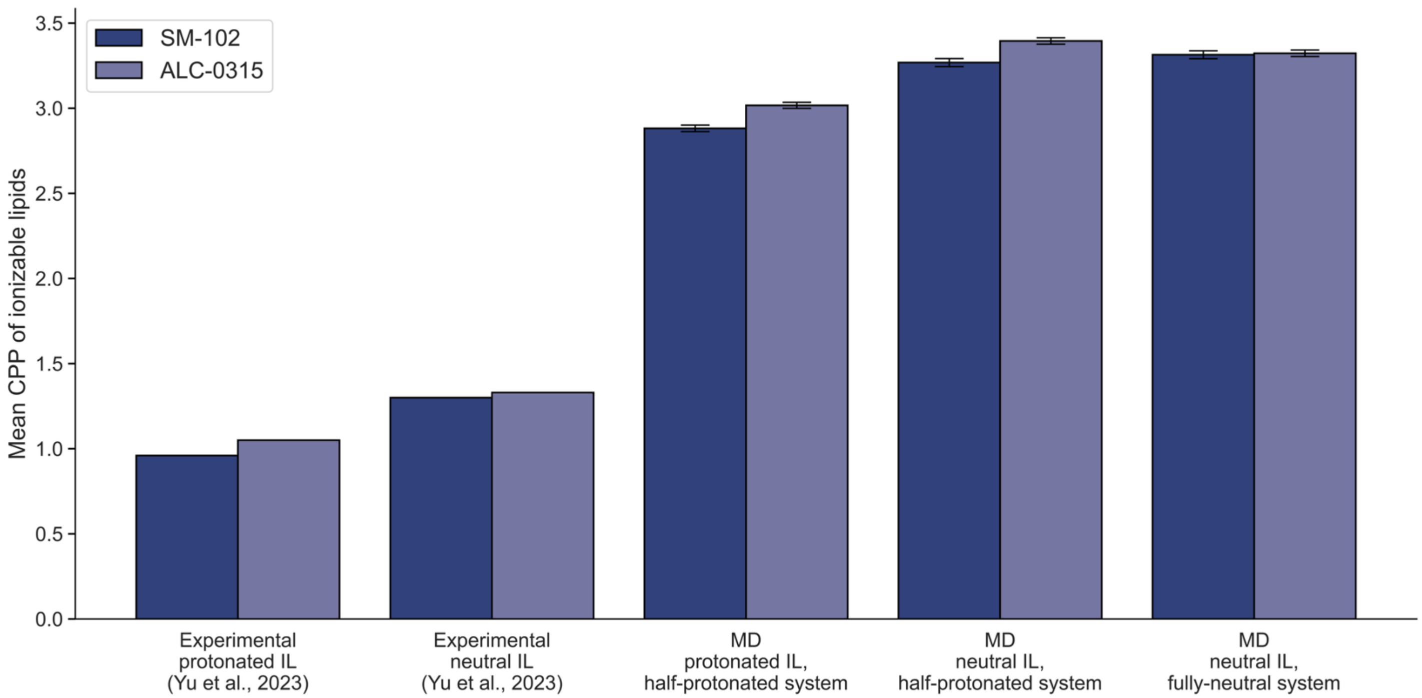

Supplementary Fig. 7: Comparison between CPP values derived experimentally and those derived from MD simulations. Experimental CPP values measured via small-angle X-ray scattering (SAXS) are derived from Yu et al., 2023[2]. MD simulations for SM-102 reflect ID “EC_13” as shown in Supplementary Table 2; MD simulations for ALC-0315 reflect ID “EC_27” as shown in Supplementary Table 2. Although overall magnitudes are different, the relative differences between SM-102 and ALC-0315 and between half-protonated and fully-neutral ionizable lipid conditions are similar between experimental and MD-derived results. This is important because capturing the relative differences in CPP between ionizable lipids – not necessarily the absolute magnitudes – is essential for accurately correlating simulation results with experimental delivery performance. Bars for MD denote mean CPP values of ionizable lipids computed as $CPP_V = V/(a_0 l_c)$, and error bars denote +/- SEM. Source data are provided as a Source data file.

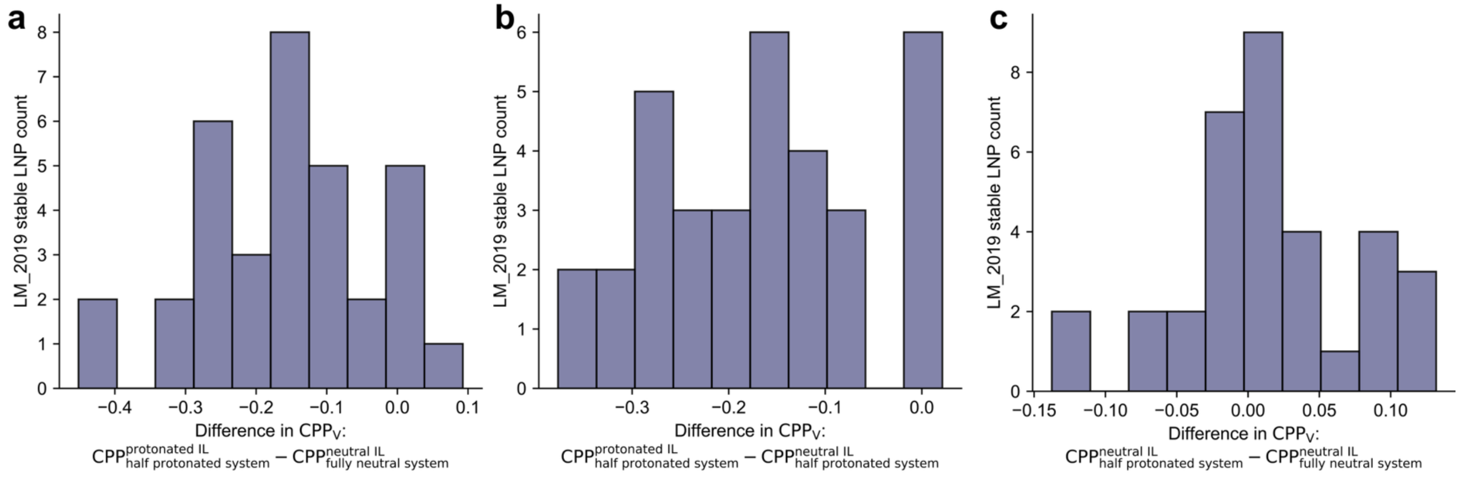

Supplementary Fig. 8: Differences in CPP values between ionizable lipids for fully-neutral and half-protonated conditions for each of the *N* = 34 LNP formulations from LM_2019 which formed stable, equilibrated bilayers. Protonated ionizable lipids tend to have lower CPP values than their respective neutral ionizable lipids, a finding which aligns with the experimental findings from Yu et al., 2023[2]. CPP values of ionizable lipids computed as $CPP_V = V/(a_0 l_c)$. Source data are provided as a Source data file.

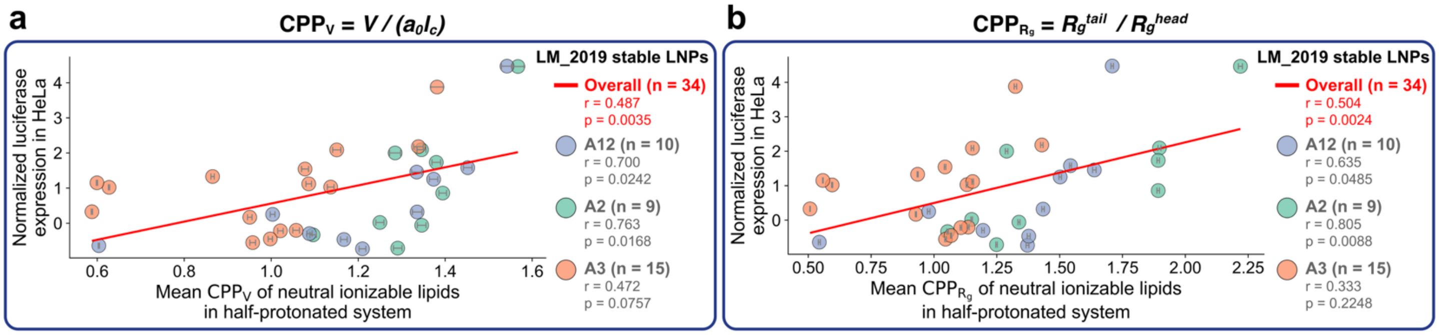

Supplementary Fig. 9: Correlation between CPP of neutral ionizable lipids and experimental delivery performance for each of the *N* = 34 LNPs from LM_2019 which formed stable, equilibrated bilayers in half-protonated systems. Neutral ionizable lipids analyzed were sourced from half-protonated systems, in contrast to neutral ionizable lipids sourced from fully-neutral systems as analyzed in Figs. 4e-f bottom. CPP values computed using **a** $CPP_V = V/(a_0 l_c)$ and **b** $CPP_{Rg} = Rg_{tail}/Rg_{head}$. Linear regression Pearson *r* and *p* values are noted for the overall trend as well as for each ionizable lipid amine group (A12, A2, A3), which are also represented by point color. Points denote mean values, and error bars denote +/- SEM for CPP_V and standard deviation for CPP_{Rg}. Source data are provided as a Source data file.

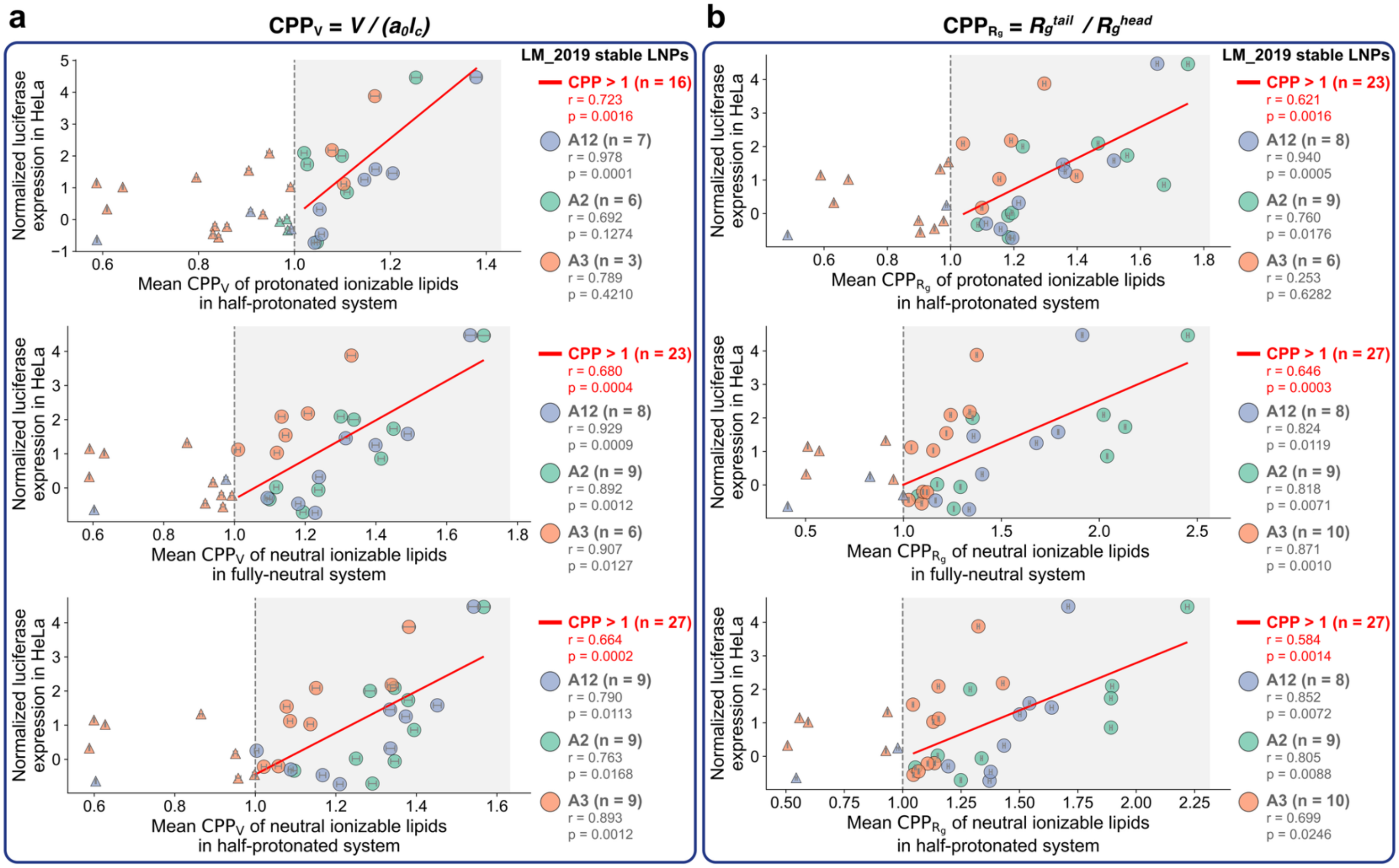

Supplementary Fig. 10: Correlation between CPP of neutral ionizable lipids and experimental delivery performance for the LNPs from LM_2019 which formed stable, equilibrated bilayers with CPP > 1. Similar analysis to Figs. 4e-f and Supplementary Fig. 9 but with correlation only computed for LNPs whose ionizable lipids have mean CPP > 1, indicating an inverse conical shape conducive to hexagonal phase formation. This CPP > 1 thresholding improves correlative performance. CPP values computed using **a** $CPP_V = V/(a_0l_c)$ and **b** $CPP_{Rg} = Rg_{tail}/Rg_{head}$. Linear regression Pearson *r* and *p* values are noted for the overall trend as well as for each ionizable lipid amine group (A12, A2, A3), which are also represented by point color. Points denote mean values, and error bars denote +/- SEM for CPP_V and standard deviation for CPP_{Rg}. Source data are provided as a Source data file.

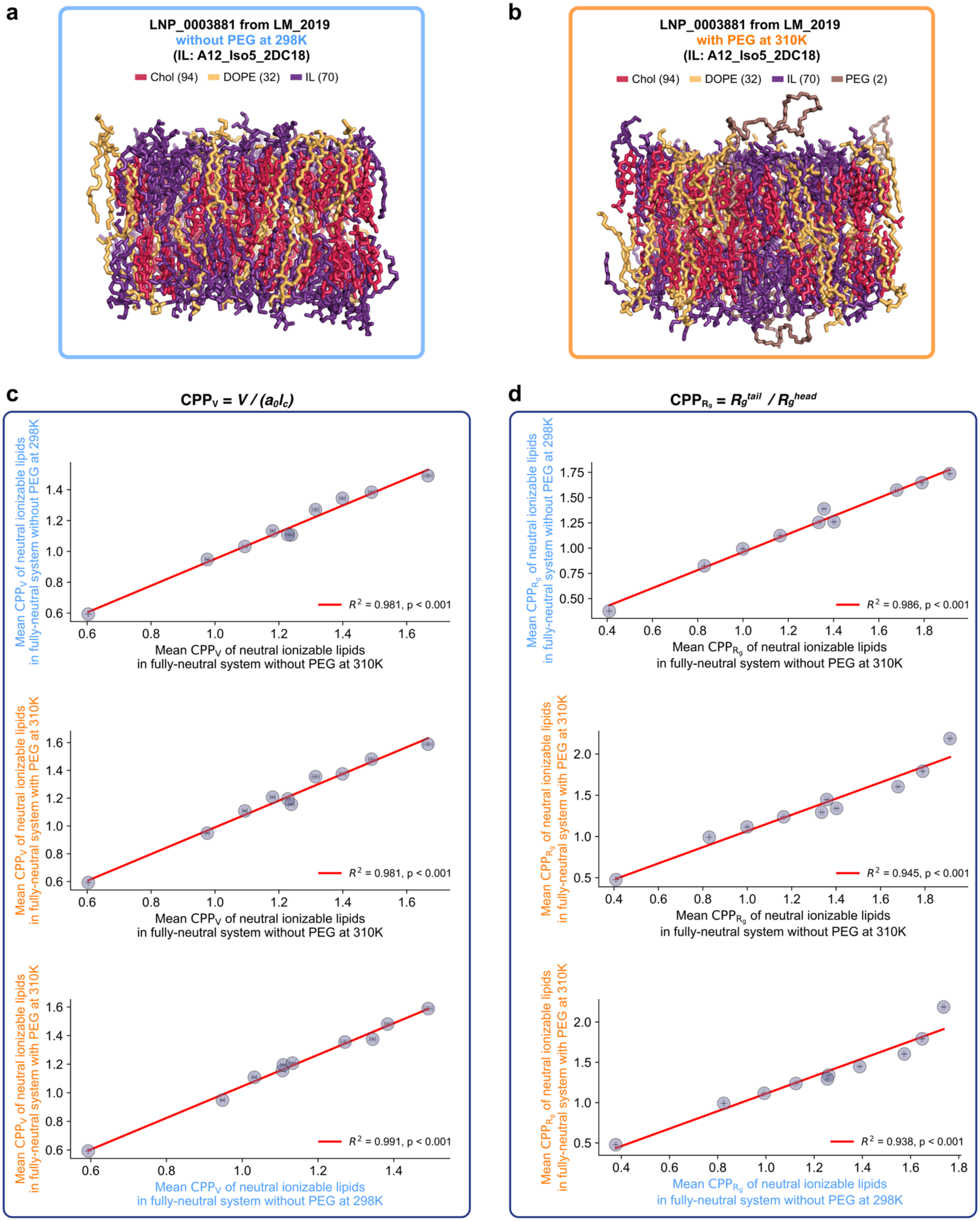

Supplementary Fig. 11: Reducing temperature to 298 K or including PEG lipid in molecular dynamics simulations does not significantly impact CPP trend. To evaluate whether reducing temperature or including PEG lipid affects CPP, 1.5 μs-long simulations were run for stable fully-neutral LM_2019 LNPs with amine 12 (n = 10), either with a reduced

temperature at 298 K or PEG lipid included. **a** Snapshot at 1.5 μs for LNP_0003881 simulated at 298 K. **b** Snapshot at 1.5 μs for LNP_0003881 with PEG lipid. PEG denotes C14 lipid with 25 monomeric PEG units. Strong correlations for **c** CPP_V and **d** CPP_{Rg} between systems without PEG at 310 K (as introduced in Fig. 4), systems without PEG at 298 K (shown in blue), and systems with PEG at 310 K (shown in orange). Source data are provided as a Source data file.

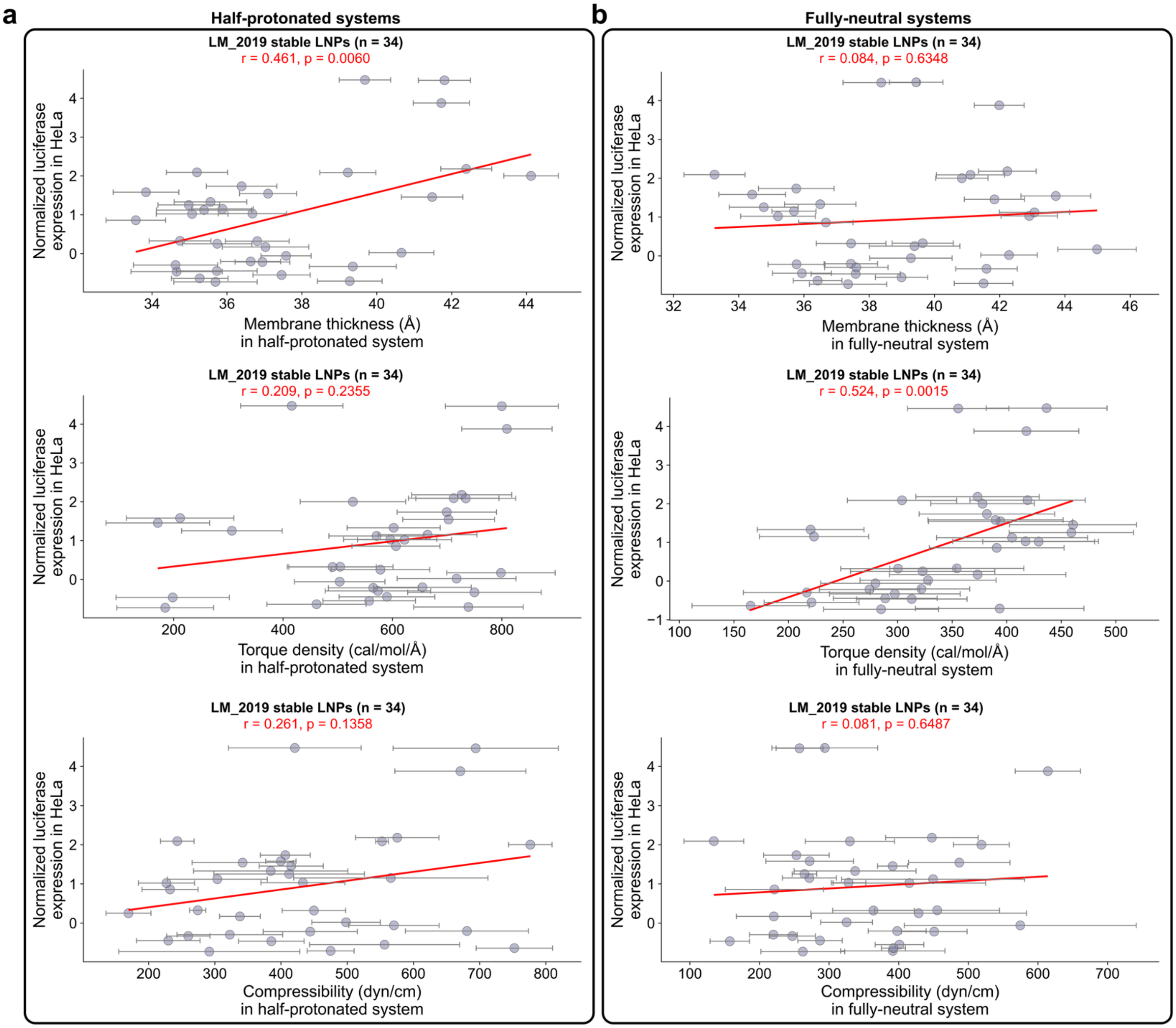

Supplementary Fig. 12: Additional bilayer metrics extracted from MD trajectories of LNP formulations from LM_2019. For both half-protonated (left) and fully-neutral (right) ionizable lipid conditions, **a** membrane thickness (Å), **b** torque density (cal/mol/Å), and **c** compressibility (dyn/cm) are calculated and assessed for correlation with experimental delivery performance. Timesteps among the final 500 ns of the 1.5 µs trajectories were analyzed. Linear regression Pearson *r* and *p* values are noted. Points denote mean values, and error bars denote +/- standard deviation. Source data are provided as a Source data file.

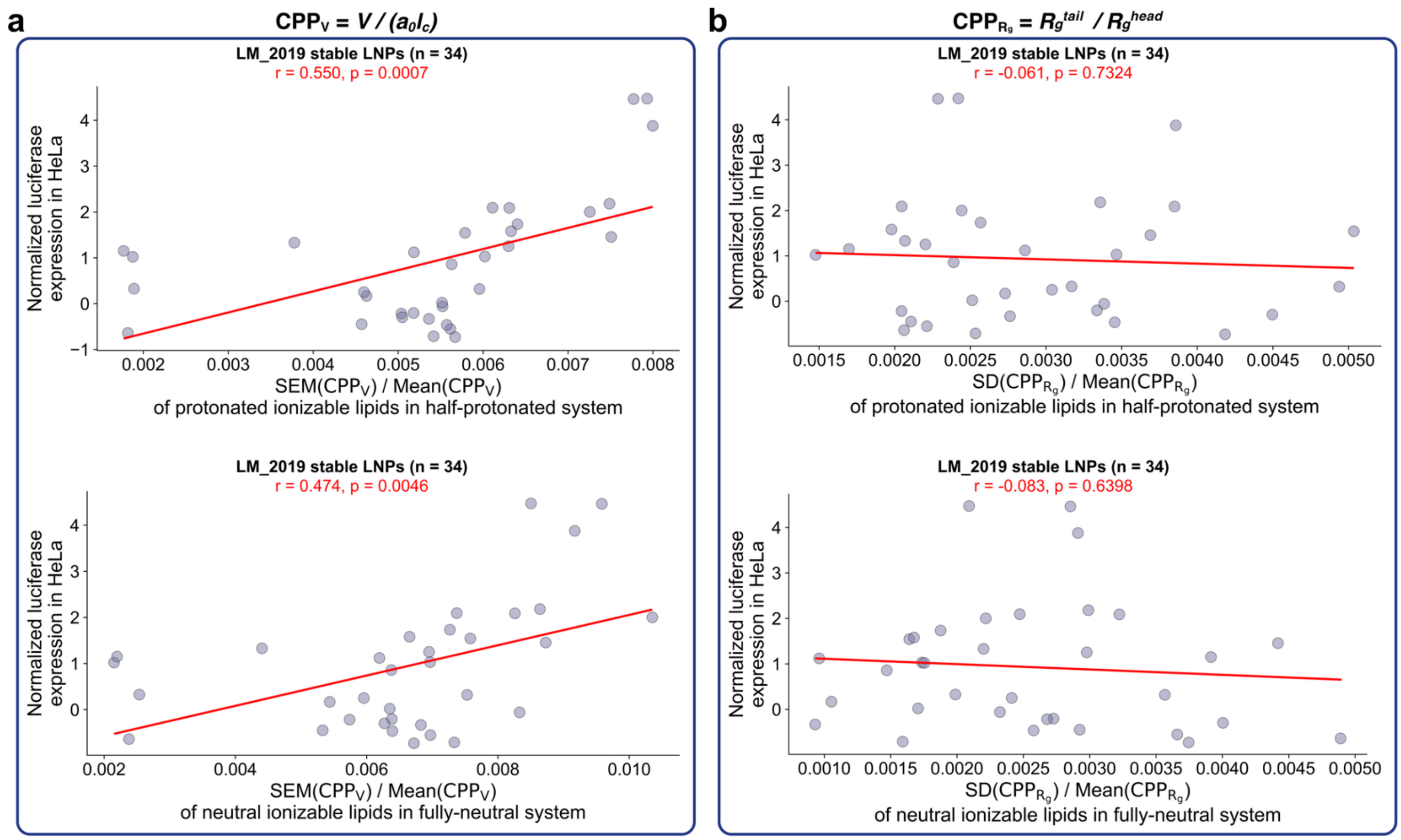

Supplementary Fig. 13. Relationship between CPP variance and delivery performance. a Analogous to Fig. 4e but assessing SEM normalized by mean CPP_V values. This significant relationship suggests greater ionizable lipid polymorphism (as measured by greater CPP_V variance) allows for more effective delivery, potentially due to increased capacity to accommodate more inverse-conical lipid geometries. **b** This relationship is insignificant for CPP_{Rg}. SEM denotes standard of the mean. SD denotes standard deviation. Linear regression Pearson *r* and *p* values are noted. Source data are provided as a Source data file.

Supplementary References

1. Miao, L. *et al.* Delivery of mRNA vaccines with heterocyclic lipids increases anti-tumor efficacy by STING-mediated immune cell activation. *Nat. Biotechnol.* **37**, 1174–1185 (2019).
2. Yu, H. *et al.* Real-Time pH-Dependent Self-Assembly of Ionisable Lipids from COVID-19 Vaccines and *In Situ* Nucleic Acid Complexation. *Angew. Chem.* **135**, e202304977 (2023).
