# Supplementary material for: Lipid Nanoparticle Database towards structure-function modeling and data-driven design for nucleic acid delivery
Source: Nat Commun. 2026 Jan 28;17:2464. doi: 10.1038/s41467-026-68818-1 (PMC12992592; doi:10.1038/s41467-026-68818-1)
Supplement: Supplementary file 2 — Reporting Summary [file 41467_2026_68818_MOESM2_ESM.pdf]

nature portfolio

Corresponding author(s): Daniel Anderson, Wonpil Im

Last updated by author(s): 01/06/2026

Reporting Summary

Nature Portfolio wishes to improve the reproducibility of the work that we publish. This form provides structure for consistency and transparency in reporting. For further information on Nature Portfolio policies, see our Editorial Policies and the Editorial Policy Checklist.

Statistics

For all statistical analyses, confirm that the following items are present in the figure legend, table legend, main text, or Methods section.

n/a	Confirmed	
☐	☒	The exact sample size (*n*) for each experimental group/condition, given as a discrete number and unit of measurement
☐	☒	A statement on whether measurements were taken from distinct samples or whether the same sample was measured repeatedly
☐	☒	The statistical test(s) used AND whether they are one- or two-sided<br>*Only common tests should be described solely by name; describe more complex techniques in the Methods section.*
☒	☐	A description of all covariates tested
☐	☒	A description of any assumptions or corrections, such as tests of normality and adjustment for multiple comparisons
☐	☒	A full description of the statistical parameters including central tendency (e.g. means) or other basic estimates (e.g. regression coefficient) AND variation (e.g. standard deviation) or associated estimates of uncertainty (e.g. confidence intervals)
☐	☒	For null hypothesis testing, the test statistic (e.g. *F*, *t*, *r*) with confidence intervals, effect sizes, degrees of freedom and *P* value noted<br>*Give P values as exact values whenever suitable.*
☒	☐	For Bayesian analysis, information on the choice of priors and Markov chain Monte Carlo settings
☒	☐	For hierarchical and complex designs, identification of the appropriate level for tests and full reporting of outcomes
☐	☒	Estimates of effect sizes (e.g. Cohen's *d*, Pearson's *r*), indicating how they were calculated

Our web collection on statistics for biologists contains articles on many of the points above.

Software and code

Policy information about availability of computer code

Data collection	To collect the data for the 19,528 LNPs featured in this initial version of LNPDB, we followed the same method as introduced in our prior study (Witten et al., Nat Biotechnol, 2024). In summary, publications were selected from the literature based on the presence of large screening datasets, primarily focused on ionizable lipids, to allow for meaningful within-dataset comparisons. Additional publications were selected to broaden representation of helper lipids, cholesterols, and PEG lipids. SMILES were created for each ionizable lipid for each publication. Functional data – most commonly delivery performance – were extracted from published heatmaps and bar plots by digitizing the figures and interpolating values based on either the color scale (heatmaps) or bar height (bar plots) as defined in the accompanying legends. Because delivery values are often reported on different scales across studies and modalities, for each publication and for each delivery context (e.g., in vivo or in vitro within the same publication), functional delivery data were standardized to have mean 0 and standard deviation 1. When raw luminescence values spanned several orders of magnitude, they were first log-transformed prior to standardization. Thus, datapoints were treated as directly comparable within individual screens, but not necessarily across different screens or assay modalities, and transformations were applied to prevent overemphasis of any single dataset.<br><br>A total of 269 ionizable lipids from the commercial supplier BroadPharm were also included in LNPDB, representing the full set available on the vendor's website as of June 1, 2024.
Data analysis	LNPDB is publicly accessible and can be interactively viewed and downloaded at https://lnpdb.molcube.com/. Source data are provided with this paper. Code used to analyze deep learning models and MD trajectories is available on our GitHub repository at https://github.com/evancollins1/LNPDB. Details about the Python package versions used can also be found on our GitHub repository.

For manuscripts utilizing custom algorithms or software that are central to the research but not yet described in published literature, software must be made available to editors and reviewers. We strongly encourage code deposition in a community repository (e.g. GitHub). See the Nature Portfolio guidelines for submitting code & software for further information.

Data

Policy information about availability of data

All manuscripts must include a data availability statement. This statement should provide the following information, where applicable:

- Accession codes, unique identifiers, or web links for publicly available datasets
- A description of any restrictions on data availability
- For clinical datasets or third party data, please ensure that the statement adheres to our policy

LNPDB is publicly accessible and can be interactively viewed and downloaded at https://lnpdb.molcube.com/. Source data for Figs. 2-4 and Supplementary Figs. 1-13 are provided with this paper.

Research involving human participants, their data, or biological material

Policy information about studies with human participants or human data. See also policy information about sex, gender (identity/presentation), and sexual orientation and race, ethnicity and racism.

Reporting on sex and gender	*Use the terms sex (biological attribute) and gender (shaped by social and cultural circumstances) carefully in order to avoid confusing both terms. Indicate if findings apply to only one sex or gender; describe whether sex and gender were considered in study design; whether sex and/or gender was determined based on self-reporting or assigned and methods used.*<br>*Provide in the source data disaggregated sex and gender data, where this information has been collected, and if consent has been obtained for sharing of individual-level data; provide overall numbers in this Reporting Summary. Please state if this information has not been collected.*<br>*Report sex- and gender-based analyses where performed, justify reasons for lack of sex- and gender-based analysis.*
Reporting on race, ethnicity, or other socially relevant groupings	*Please specify the socially constructed or socially relevant categorization variable(s) used in your manuscript and explain why they were used. Please note that such variables should not be used as proxies for other socially constructed/relevant variables (for example, race or ethnicity should not be used as a proxy for socioeconomic status).*<br>*Provide clear definitions of the relevant terms used, how they were provided (by the participants/respondents, the researchers, or third parties), and the method(s) used to classify people into the different categories (e.g. self-report, census or administrative data, social media data, etc.)*<br>*Please provide details about how you controlled for confounding variables in your analyses.*
Population characteristics	*Describe the covariate-relevant population characteristics of the human research participants (e.g. age, genotypic information, past and current diagnosis and treatment categories). If you filled out the behavioural & social sciences study design questions and have nothing to add here, write "See above."*
Recruitment	*Describe how participants were recruited. Outline any potential self-selection bias or other biases that may be present and how these are likely to impact results.*
Ethics oversight	*Identify the organization(s) that approved the study protocol.*

Note that full information on the approval of the study protocol must also be provided in the manuscript.

Field-specific reporting

Please select the one below that is the best fit for your research. If you are not sure, read the appropriate sections before making your selection.

☒ Life sciences ☐ Behavioural & social sciences ☐ Ecological, evolutionary & environmental sciences

For a reference copy of the document with all sections, see nature.com/documents/nr-reporting-summary-flat.pdf

Life sciences study design

All studies must disclose on these points even when the disclosure is negative.

Sample size	To assess whether MD simulations could provide meaningful correlations with experimental delivery performance (Figs. 4b-e), we selected N = 54 LNP formulations from a prior study (LM_2019) from our lab which introduced a isocyanide-mediated three-component reaction approach for ionizable lipids. For the sake of modeling, we randomly selected to model the subset of LNPs that contain ionizable lipids with amines A12, A2, or A3; isocyanides Iso5 or Iso9; and any alkyl ketone, as shown in Fig. 1c of our prior study. PEG lipids were excluded from simulations, as they are typically shed prior to endosomal escape, the key bottleneck for effective delivery and the physiological context that we aimed to model. This subset, drawn from a single combinatorial ionizable lipid library, was chosen as a representative example of systematic lipid library design commonly employed in the field, while keeping the scope feasible within computational limits.<br><br>Additional simulations were conducted for illustrative purposes (Figs. 1b, 4a-b) that contain PEG lipid or the common control ionizable lipids of DLin-MC3-DMA, SM-102, and ALC-0315. Details of all bilayer simulations analyzed in this study are provided in Table S2.
Data exclusions	In Fig. 3a, some datasets shared in part between LNPDB and the original dataset from our prior paper (Witten et al., Nat Biotechnol, 2024) were excluded from performance comparison for different data-quality reasons: AA_2008 lacks helper lipid, LL_2012 and LR_2023 contained

	discretized data which skewed performance, JM_2016 had low sample size, and the data of JW_2024 was expanded compared to our prior so denotes different datasets now.
Replication	Code provided in GitHub to replicate deep learning models and CPP analysis of MD trajectories.
Randomization	Not relevant.
Blinding	Not relevant.

Reporting for specific materials, systems and methods

We require information from authors about some types of materials, experimental systems and methods used in many studies. Here, indicate whether each material, system or method listed is relevant to your study. If you are not sure if a list item applies to your research, read the appropriate section before selecting a response.

Materials & experimental systems

n/a	Involved in the study
☒	☐ Antibodies
☒	☐ Eukaryotic cell lines
☒	☐ Palaeontology and archaeology
☒	☐ Animals and other organisms
☒	☐ Clinical data
☒	☐ Dual use research of concern
☒	☐ Plants

Methods

n/a	Involved in the study
☒	☐ ChIP-seq
☒	☐ Flow cytometry
☒	☐ MRI-based neuroimaging

Plants

Seed stocks	*Report on the source of all seed stocks or other plant material used. If applicable, state the seed stock centre and catalogue number. If plant specimens were collected from the field, describe the collection location, date and sampling procedures.*
Novel plant genotypes	*Describe the methods by which all novel plant genotypes were produced. This includes those generated by transgenic approaches, gene editing, chemical/radiation-based mutagenesis and hybridization. For transgenic lines, describe the transformation method, the number of independent lines analyzed and the generation upon which experiments were performed. For gene-edited lines, describe the editor used, the endogenous sequence targeted for editing, the targeting guide RNA sequence (if applicable) and how the editor was applied.*
Authentication	*Describe any authentication procedures for each seed stock used or novel genotype generated. Describe any experiments used to assess the effect of a mutation and, where applicable, how potential secondary effects (e.g. second site T-DNA insertions, mosiacism, off-target gene editing) were examined.*
